# Metal-Organic-Framework FeBDC-Derived Fe_3_O_4_ for Non-Enzymatic Electrochemical Detection of Glucose

**DOI:** 10.3390/s20174891

**Published:** 2020-08-29

**Authors:** Syauqi Abdurrahman Abrori, Ni Luh Wulan Septiani, Nugraha, Isa Anshori, Suyatman, Veinardi Suendo, Brian Yuliarto

**Affiliations:** 1 Advanced Functional Materials Laboratory, Engineering Physics Department, Faculty of Industrial Technology, Bandung Institute of Technology, Bandung 40132, Indonesia; abdsyauqi@gmail.com (S.A.A.); niluhwulan@gmail.com (N.L.W.S.); nugraha@tf.itb.ac.id (N.); 2 Research Center for Nanosciences and Nanotechnology (RCNN), Bandung Institute of Technology, Bandung 40132, Indonesia; isa_anshori@stei.itb.ac.id (I.A.); vsuendo@mail.chem.itb.ac.id (V.S.); 3 Lab-on-Chip Group, Biomedical Engineering Department, Bandung Institute of Technology, Bandung 40132, Indonesia; 4 Division of Inorganic and Physical Chemistry, Faculty of Mathematics and Natural Sciences, Bandung Institute of Technology, Bandung 40132, Indonesia

**Keywords:** FeBDC, Fe_3_O_4_, glucose, electrochemical, non-enzymatic glucose

## Abstract

Present-day science indicates that developing sensors with excellent sensitivity and selectivity for detecting early signs of diseases is highly desirable. Electrochemical sensors offer a method for detecting diseases that are simpler, faster, and more accurate than conventional laboratory analysis methods. Primarily, exploiting non-noble-metal nanomaterials with excellent conductivity and large surface area is still an area of active research due to its highly sensitive and selective catalysts for electrochemical detection in enzyme-free sensors. In this research, we successfully fabricate Metal-Organic Framework (MOF) FeBDC-derived Fe_3_O_4_ for non-enzymatic electrochemical detection of glucose. FeBDC synthesis was carried out using the solvothermal method. FeCl_2_.4H_2_O and Benzene-1,4-dicarboxylic acid (H_2_BDC) are used as precursors to form FeBDC. The materials were further characterized utilizing X-ray Powder Diffraction (XRD), Scanning Electron Microscopy (SEM), and Fourier-Transform Infrared Spectroscopy (FTIR). The resulting MOF yields good crystallinity and micro-rod like morphology. Electrochemical properties were tested using Cyclic Voltammetry (CV) and Differential Pulse Voltammetry (DPV) with a 0.1 M of Phosphate Buffer Saline (PBS pH 7.4) solution as the supporting electrolyte. The measurement results show the reduction and oxidation peaks in the CV curve of FeBDC, as well as Fe_3_O_4_. Pyrolysis of FeBDC to Fe_3_O_4_ increases the peak of oxidation and reduction currents. The Fe_3_O_4_ sample obtained has a sensitivity of 4.67 µA mM^−1^.cm^−2^, a linear range between 0.0 to 9.0 mM, and a glucose detection limit of 15.70 µM.

## 1. Introduction

The dire need for rapid and reliable glucose monitoring has grown to be a developing concern over the past few years in food industries, biomedical, and clinical diagnostics. The highest necessity is resulting from diabetes mellitus, a disease caused by abnormalities of blood sugar (glucose) level that impacts tens of millions of people everywhere in the world [1,2,3]. Diabetes is a chronic disease that occurs when the body’s production of insulin is insufficient (type 1 diabetes) or when the insulin in the human body is ineffective (type 2 diabetes). In principle, insulin works by converting glucose or blood sugar into glycogen. If insulin cannot work correctly, it will cause a buildup of glucose in the blood [4]. High glucose level can cause severe damage to the heart, blood vessels, eyes, kidneys, and nerves. Therefore, it is essential to measure glucose levels in the body routinely to prevent the accumulation of glucose in the blood, which can cause diabetes [5]. This challenge leads to numerous research and innovation that provide various methods for glucose detection, including fluorescent, optical, colorimetry, chromatographic, acoustic, and electrochemical [2,6,7,8,9,10]. Among the methods mentioned above, the most cost-effective approach would be the electrochemical technique due to its excellent sensitivity, easy operational, simple instrumentation, and affordable cost [11,12,13,14]. The electrochemical technique can be broadly categorized into two main categories, enzymatic and non-enzymatic, where enzymatic biosensors involve the use of enzymes to detect analytes, including glucose. Some enzymes that can be used to detect glucose include glucose dehydrogenase and glucose oxidase. Contrastly, the non-enzymatic biosensors do not require any enzymes and the detection principle is based on direct oxidation of the analytes on the surface of the sensing layer [15].

Despite its sensitivity and selectivity, enzymatic biosensors suffer from various limitations due to the characteristics of the enzyme that is easy denaturation, inferior stability, and costly. Furthermore, various environmental factors such as pH, humidity, temperature, organic reagents, and toxic chemicals can easily affect the activity of enzymes. This affected enzyme activity results in lower sensitivity and reproducibility of the fabricated enzymatic biosensors [2,15,16]. These drawbacks were easily overcome by the non-enzymatic biosensor, owing to its numerous traits such as its excellent stability, reproducibility, simplicity, and good selectivity, which make non-enzymatic biosensor an appealing and reliable instrument for glucose monitoring [13,17,18].

A sensitive and selective electrocatalyst is highly necessary to produce an exceptional non-enzymatic biosensors as it can significantly affect the oxidation process of the analyte directly with minimum resistance, the electrochemical reaction that occurs at the electrolyte/electrode interface, and the conduction of electrons in the electrode [19,20]. However, there are several obstacles to the oxidation of glucose in the non-enzymatic biosensors; one of those obstacles is that this process usually requires relatively high over-potential in conventional electrodes. One of the strategies to overcome this obstacle is by modifying the electrodes using metal nanocatalysts. Noble-metal-based nanocatalysts, including platinum (Pt), gold (Au), silver (Ag), and palladium (Pd)-based nanocatalyst, have been widely studied as electrocatalytic materials for the non-enzymatic electrochemical oxidation. Even though those noble metal nanocatalysts are considered as effective electrocatalysts towards electrochemical oxidation of glucose, they still have several shortcomings, including limited sensitivity, poor selectivity, and narrow linear ranges, owing to the interferences from chloride ions and the surface poisoning/fouling from the adsorbed intermediates. Furthermore, the high cost and low abundance of those noble metal catalysts do not make this approach a good alternative for large scale applications [21].

Recently, many glucose sensors were utilizing the more abundant and inexpensive transition metals and their oxide structures such as Ni, Co, Cu, NiO, Co_3_O_4_, CuO, ZnO, MnO_2_, Mn_3_O_4_, WO_3_, TiO_2_, CdO, and Fe_2_O_3_ [21]. These catalysts can be used for non-enzymatic electrochemical detection of glucose due to its superior catalytic activity, large surface area, abundant availability, ease of synthesis, and low cost [22,23]. Among these materials, various structures of ferrous iron-oxide like ferrihydrite, hematite, and magnetite, have been made into electrochemical electrodes to detect glucose non-enzymatically. The utilization of iron-oxide nanostructures is due to several characteristics, including large specific surface area, sensitive and selective electrocatalytic activity, and biocompatibility [22,24,25,26]. For instance, Liu et al. [27] used hierarchical α-Fe_2_O_3_ microcubes supported on Ni foam. They successfully measured glucose concentrations in PBS (pH 7.4) of 0.1 M. The sensor performance was as good as most of the other transition metal-based sensors (linear range of 0.005–0.2 mM, the limit of detection = 0.87 μM, sensitivity = 10,356 μA mM^−1^cm^−2^, response time < 3 s, excellent stability and selectivity in the presence of uric acid (UA), dopamine (DA), ascorbic acid (AA), NaCl, cysteine (Cys), and other interferences).

Furthermore, Fe-based MOFs with the coordination of metal clusters and carboxylic ligands by various covalent bonds and intermolecular forces have been synthesized and applied for emergent material fields [28]. Typically, a hybrid FeBDC (BDC = benzene-1,4-dicarboxylate) exhibited high porosity as well as promising physical/chemical properties for membranes, coatings, sensors, gas storage, and drug delivery [29,30]. Bhattacharjee et al. has conducted a study on FeBDC, which was synthesized using the solvothermal method [31]. The results of this study indicate that the making of FeBDC using the solvothermal method is relatively easy. The resulting FeBDC has high porosity, and the electrochemical response obtained shows promising results. These characteristics make FeBDC a potential material for research and development as an active glucose sensor material. Moreover, due to highly active open metal sites of Fe-based MOFs, many efforts orientated new utilizations [32,33,34,35]. The principle for the strategies rely on the transformation of MOFs into metals/metal oxides and organic matters for the advent of functional groups; thus, this functionalization process aids surface modification [36]. Under the controllable thermolysis conditions, volatile molecules, including carbon oxides vapor, can be rapidly released from the mother precursors, then, leaving a porously hollow nanostructured residue [37]. Those works have attempted to extend the potentials of Fe-MOFs to electrochemical glucose sensing applications. In this work, we used Fe_3_O_4_, obtained from pyrolyzing of FeBDC, as a sensitive layer for non-enzymatic electrochemical glucose sensors. Differential Pulse Voltammetry (DPV) technique was employed to investigate the effect of the glucose molecules on the surface magnetite properties.

## 2. Materials and Methods

Iron (II) chloride tetrahydrate (FeCl_2_.4H_2_O), Terephthalate acid (H_2_BDC), N,N-dimethylformamide (DMF), glucose, Nafion, and other common reagents were acquired from Sigma-Aldrich, Singapore. The organic solutions such as 2-propanol (IPA), methanol, and ethanol were purchased from Merck, Singapore. All chemicals were of analytical grade and used without any further purification.

FeBDC was prepared by a simple solvothermal method following a procedure reported in the literature [31]. Typically, 0.332 g of H_2_BDC (2 mmol) was dissolved in a mixture of 37 mL of DMF, 2 mL of IPA, and 2 mL of distilled water and then stirred using a magnetic stirrer for 15 min at a speed of 300 rpm. After dissolving, the FeCl_2_.4H_2_O precursor with a mass of 0.795 g (4 mmol) is slowly added to the solution under vigorous stirring until all the precursors are entirely dissolved. The mixture solution is then placed in a 100 mL Teflon stainless autoclave and heated in a furnace at 105 °C for 24 h. The result of dark brown FeBDC powder afterward was washed using DMF, methanol, and ethanol three times for each solvent so that the pores of FeBDC could be thoroughly cleaned. The last step is drying at 80 °C for 12 h, and the FeBDC powder has been obtained. To obtain Fe_3_O_4_, the resulted FeBDC was pyrolyzed under the N_2_ atmosphere. The temperature was set from room temperature to 500 °C and hold it for 120 min.

Bruker D8 Advanced collected the X-ray Diffraction (XRD) patterns with CuKα x-ray generator, λ = 1.5418 Å. The morphologies property of the MOF and Fe_3_O_4_ were analyzed using Scanning Electron Microscopy (SEM) of the Hitachi SU3500 series with a resolution of 10 nm at the accelerating voltage of 5 kV. Fourier Transform Infrared (FTIR) spectra were recorded by Prestige 21 Shimadzu with a resolution of 2 cm^−1^. The thermogravimetry and Differential Thermal Analysis (TG/DTA) was carried out using Hitachi 7300 Simultaneous Thermogravimetric Analyzer, at a temperature in the range of RT-600 °C under N_2_ atmosphere and heating rate of 10 °C/min.

**Figure 1:**
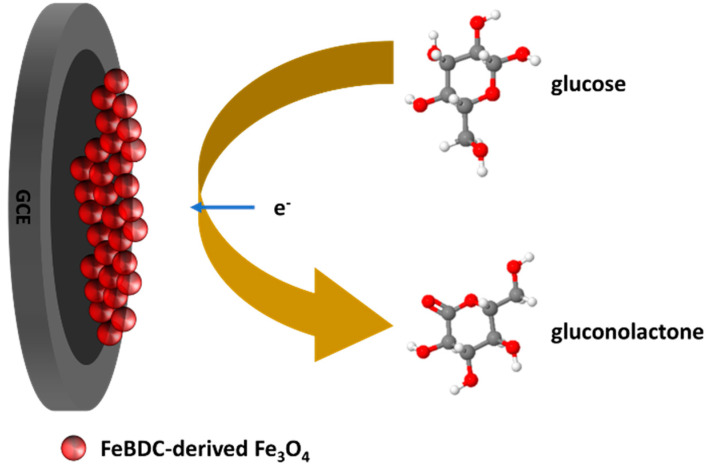
Schematic illustration of the glucose oxidation on the Fe_3_O_4_/GCE.

The electrochemical sensing mechanism of glucose by FeBDC or Fe_3_O_4_ is illustrated in Figure 1. All electrochemical measurements were carried out in a standard three-electrode system by CorrTest CS Series Electrochemical Station at room temperature and in an aerated condition. The three-electrode system consists of a working electrode, a counter electrode, and a reference electrode. The working electrode, which makes contact with the analyte, is an electrode that is modified with the sample material. The role of the reference electrode is to act as a reference in measuring and controlling the working electrode potential, without passing any current. Moreover, the reference electrode is a half cell with a known reduction potential. Meanwhile, the role of the counter electrode is to complement the working electrode to form a perfect circuit.

In this work, we employed a glassy carbon electrode (GCE, 3 mm in diameter) modified with the samples as the working electrode. Meanwhile, a Pt wire was used as the counter electrode, and an Ag/AgCl standard electrode (3 M KCl) was used as the reference electrode. Phosphate Buffer Saline (PBS) solution with pH 7.4 and a concentration of 0.1 M was employed as the supporting electrolyte for non-enzymatic glucose sensing. To prepare the Fe_3_O_4_-modified GCEs, 5 mg ofFe_3_O_4_ powder was dispersed into 1 mL of ethanol and sonicated for 15 min to form a uniform suspension. Then, 10 μL of Fe_3_O_4_ suspension (5 mg/mL) and 5 μL of Nafion (0.5 wt%) were drop cast onto the polished surface of GCE and dried under ambient temperature. Afterward, the three-electrodes are exposed to the analytes solution with pre-determined concentration. The Cyclic Voltammograms (CV) of the samples were carried out in the potential range of 0–1.0 V versus the Ag/AgCl standard reference electrode with a scan rate of 50 mV/s. Meanwhile, DVP was carried out in 0.1 M PBS pH 7.4 over the potential range of 0.0–1.0 V with a step, pulse amplitude, pulse width, and pulse period of 4 mV, 0.05 V, 0.05 s, and 2 s, respectively.

## 3. Results and Discussion

### 3.1. Structural Characterization

**Figure 2:**
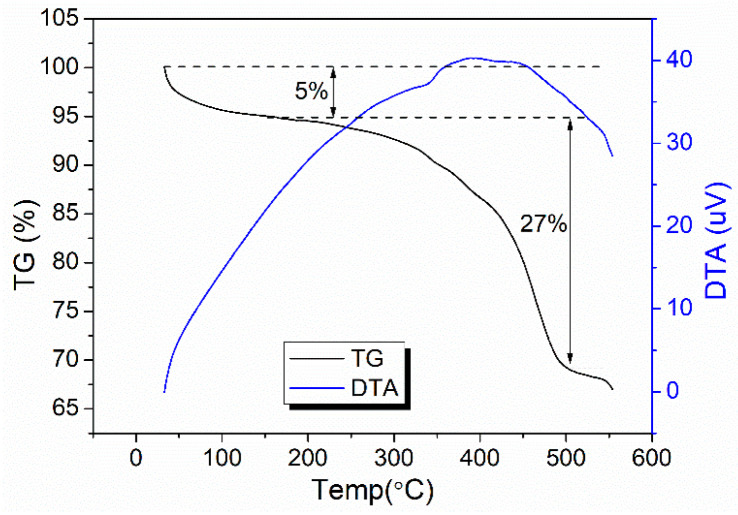
Thermogravimetry/Differential Thermal Analysis (TG/DTA) curve of MOF FeBDC.

The FeBDC MOF powder will be used as a template of iron oxide material by pyrolyzing at 500 °C under a nitrogen atmosphere for 120 min. The pyrolisis was carried out at the temperature of 500 °C based on the results of the TG/DTA of the MOF FeBDC material. TG/DTA aims to characterize the thermal properties of the material based on the material’s response to temperature. The results of the TG/DTA thermal analysis are used to determine the pyrolysis temperature required by FeBDC MOF to be able to become Fe_3_O_4_ entirely. The TG/DTA analysis result can be seen in Figure 2.

From Figure 2, it appears that the MOF of FeBDC experienced two-steps of mass reduction, at temperatures around 100–200 °C and 350–450 °C. The decrease in mass that occurs at the temperature of 100–200 °C was caused by DMF solvents’ evaporation that is still contained in the FeBDC MOF powder. At 350–450 °C, a relatively significant mass loss was possibly caused by the decomposition of the hydroxyl group on the MOF surface and excessive water, followed by decomposition of BDC linker that was coordinated with metal ion core [38,39,40]. Besides, the transformation reaction involving recrystallization was indicated by an exothermic peak in the DTA curve. The recrystallization process occurred slowly, where the process rate is slower than the heating rate (in this case, TG/DTA was carried out with the heating rate of 10 °C/min) and cause broadening exotherm peak to higher temperature [41]. In other words, the recrystallization cannot be completed at a specific temperature, and expand to a higher temperature, resulting in a broad DTA peak. The pyrolysis of FeBDC to Fe_3_O_4_ is carried out at 500 °C because at 500 °C, the organic ligands have entirely decomposed, and the Fe-BDC was entirely converted to Fe_3_O_4_.

**Figure 3:**
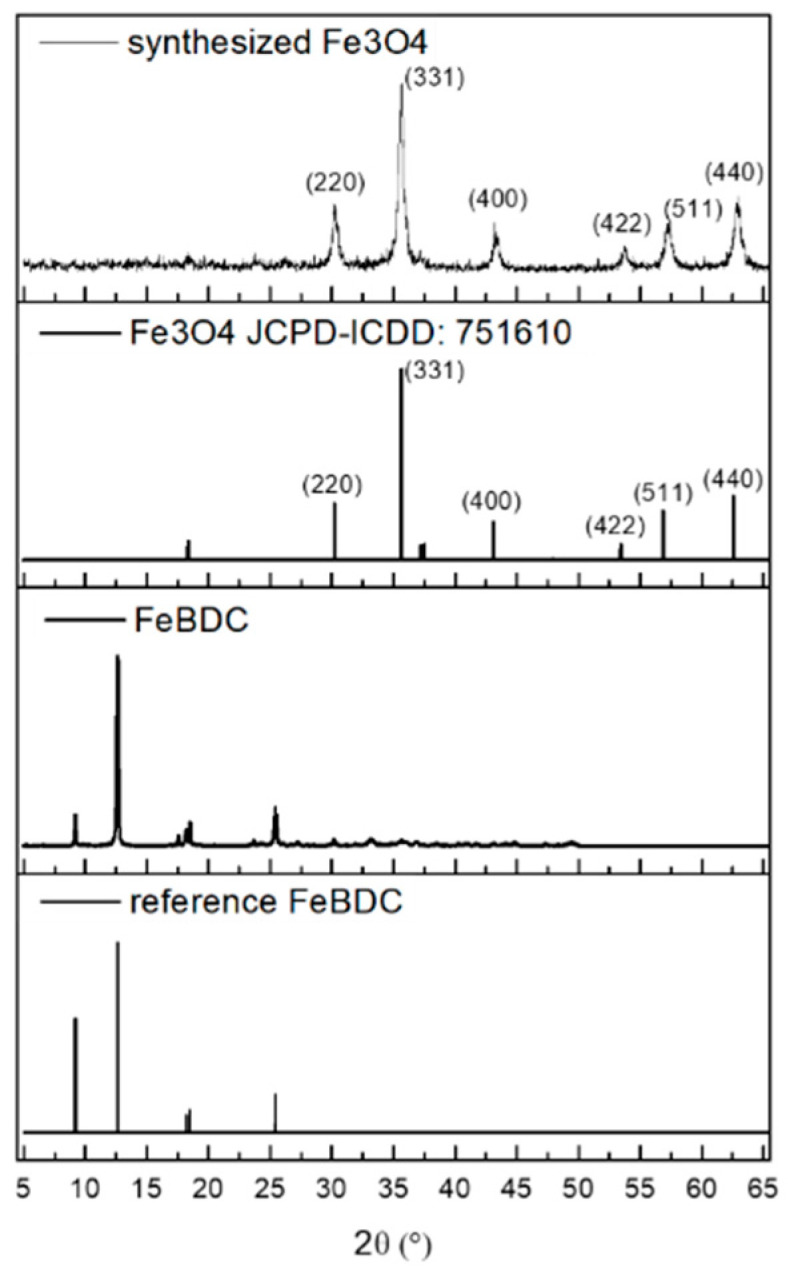
X-ray Diffraction (XRD) spectra of FeBDC, Fe_3_O_4_, and their respective references.

XRD is one method of material characterization which results in the form of diffraction peak patterns associated with crystallinity formed in the samples. The XRD diffraction pattern of FeBDC resulting from the synthesis and its oxide derivative is shown in Figure 3. Based on the displayed Fe-BDC pattern, a sharp diffraction peak at 2θ = 9.2°; 12.6°; 17.6°; 18.2°; 18.5°; and 25.4° are typical peaks of Fe-BDC and match well with the Cambridge Crystallographic Data Centre (CCDC) card No. 690315 [40]. That diffraction pattern is in accordance with the work of [39,40,41,42]. Whereas Figure 3 also shows the diffraction pattern of Fe_3_O_4_ metal oxide, which is the result of MOF FeBDC pyrolyzed at 500 °C for 1 h. The XRD peaks match well with the characteristic peaks of the inverse cubic spinel structure crystalline structure of Fe_3_O_4_ in the International Centre for Diffraction Data (JCPDS-ICDD: 751610).

Moreover, there is no impurity observed in the XRD pattern of Fe_3_O_4,_ indicating the complete transformation of FeBDC to Fe_3_O_4_ as well as complete decomposition of the organic compound. The average crystallite size d is calculated using the Debye–Scherrer equation d = Kλ/(βcosθ), where K is Scherrer constant, in this case, we used 0.9, λ is X-ray wavelength, β is full width at half-maximum (FWHM) of the XRD peak, and θ is Bragg angle. The calculated average crystallite size is 17.3 nm and this is lower than Fe_3_O_4_ synthesized by Masoomi-Godarzi et al. (2013) [17].

**Figure 4:**
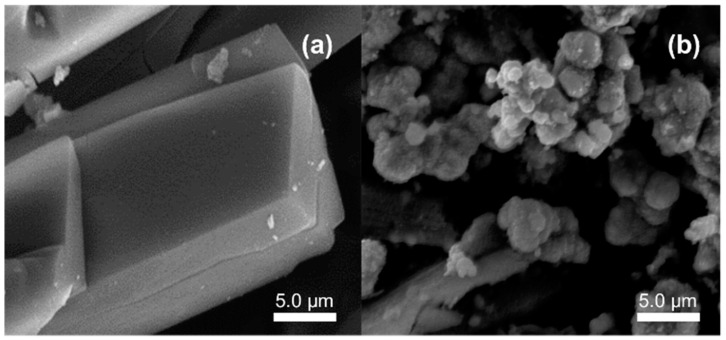
Scanning Electron Microscopy (SEM)images of (**a**) FeBDC (**b**) FeBDC-derived Fe_3_O_4_.

**Figure 5:**
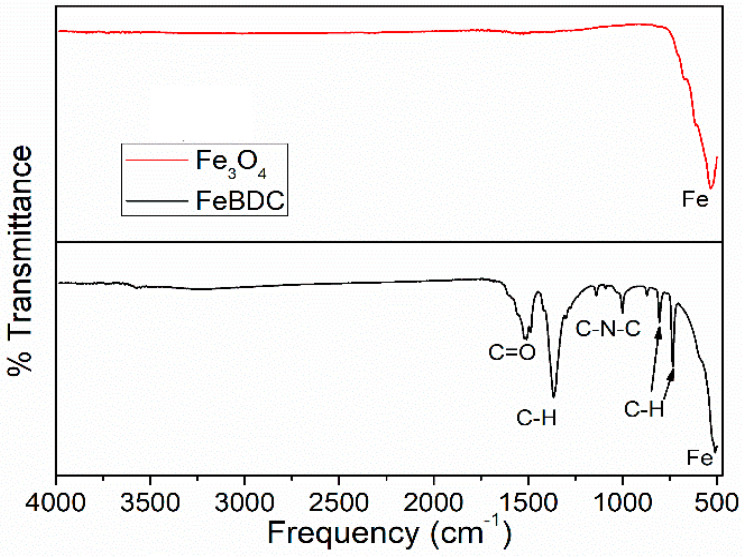
Fourier Transform Infra Red (FTIR) spectra of FeBDC and Fe_3_O_4_.

SEM analysis was performed to determine the surface morphology characteristics of a material. Figure 4a shows SEM images from the synthesized FeBDC MOF. It shows that the FeBDC MOF has a particle shape, such as the FeBDC MOF produced by Liu et al. in 2017 [43]. The solvothermal method at 105 °C drove the formation of bulky triangular rodlike FeBDC with an average size of 20 µm. Similar bulky rodlike particles have been observed by other works indicating the typical shape of FeBDC [44]. It has been reported that the oxidation state of the Fe source affects the resulted shape and size. Small bulky particles transformed into bigger bulky-rodlike shape particle when FeCl_3_ replace to FeCl_2_. The use of FeCl_2_ in this work is in agreement with the previous report [44]. The coordination between oxygen atom from DMF solvent and Fe (II) forming FeO_6_ octahedral and BDC-bridging results in crystal growth in one dimensional [45]. Based on the two test results, XRD and SEM test, it can be concluded that the FeBDC MOF has been successfully synthesized using the solvothermal method because the crystallinity and morphology are in accordance with the reference FeBDC. However, FeBDC derived Fe_3_O_4_ is found to be very different from that of FeBDC, indicating the MOF cannot maintain its shape during heat treatment. The converted Fe_3_O_4_ has an irregular spherical shape with the size in the range of 1–3 µm. The decomposition of the organic linker causes the structure to be collapsed to smaller particles. Indeed, Figure 4b shows a noticeable deficiency in crystallinity, trending an amorphous nature of Fe_3_O_4_ with a strongly defective surface. The remarkable change in morphological properties between FeBDC and Fe_3_O_4_ can lead to a respective movement of functional group bonds in the structure, which could also be supported by the FTIR technique in Figure 5.

Functional groups involved in organic or inorganic materials can be identified by FTIR. Figure 5 shows the FTIR spectroscopic results of the MOF FeBDC material and Fe_3_O_4_. The absence of broad peaks in the range of 3000–4000 cm^−1^ indicates that there is no O-H stretching vibration originating from water vapor, and it could also mean that FeBDC is stable in a humid environment. Sharp peaks at 1538 cm^−1^ and 1525 cm^−1^ are indicating the presence of C=O stretching vibration and -COO- asymmetric stretching vibrations, respectively. Moreover, the bending vibration of C-H in -CH_3_ is attributed to the relatively sharp peak at 1382 cm^−1^. A small peak at 1016 cm^−1^ is assigned to the C-N-C vibration bond in the BDC linker.

Additionally, the peaks at 821 cm^−1^ and 751 cm^−1^ are assigned to C-H bending vibration in the benzene ring of BDC, while the peak at 527 cm^−1^ in the fingerprint region indicated the Fe-O bond of Fe with the BDC linker through oxygen site [40,46,47]. However, the peaks attributed to the BDC bonds disappear when the pyrolysis process is carried out to obtain metal oxide Fe_3_O_4_. This disappearance indicates that the BDC ligands in the MOF were utterly decomposed, and the material has been completely transformed into iron oxide Fe_3_O_4_.

### 3.2. Electrochemical Sensor Characterization

**Figure 6:**
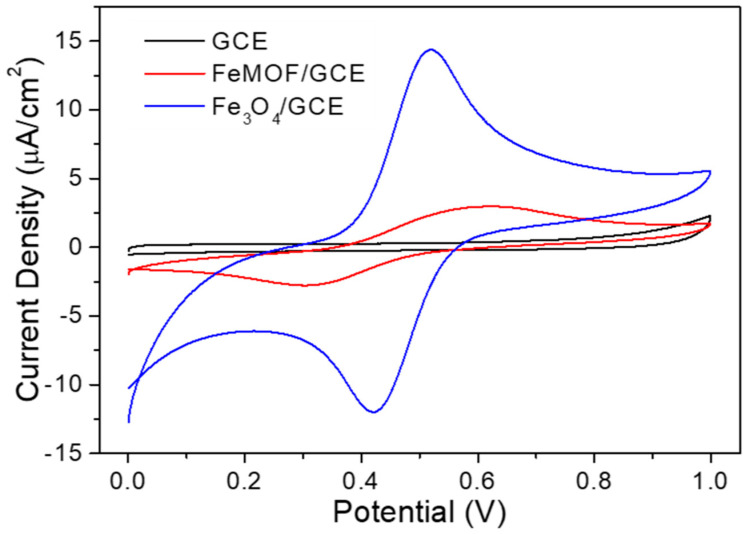
Cyclic Voltamogram (CV) spectra of bare GCE, FeBDC/GCE. Fe_3_O_4_/GCE in 3 mM of glucose and Phosphate Buffer Saline (PBS) of 0.1 M pH 7.4.

Herein we determine the electrochemical properties of synthesized FeBDC and Fe_3_O_4_ using cyclic voltammetry (CV) measurement. Figure 6 shows the CV curve results of MOF FeBDC and Fe_3_O_4_ deposited on GCE compared to GCE without any modification or bare GCE. The CV test was carried out in the presence of glucose with a concentration of 3 mM in 0.1 M of PBS pH 7.4 and measured within the potential window from 0 to 1.0 V (vs. commercial Ag/AgCl reference electrode). From Figure 6, it can be seen that the GCE with FeBDC modification shows the peak of the oxidation current at a potential of 0.625 V and the peak of the reduction current at a potential of 0.304 V while the bare GCE does not show any peak represent of the oxidation or reduction current. The results of current measurements and oxidation and reduction potential are tabulated in Table 1. From the Table 1, it can be seen that the FeBDC material can increase the oxidation current at a potential of 0.625 V by 764% and the reduction current by 989% at a potential of 0.304 V when compared to the oxidation and reduction currents at the same potential from bare GCE. In other words, the FeBDC and Fe_3_O_4_ are catalytically active as glucose sensor materials.

From Figure 6 and Table 1, it can be seen that the pyrolysis of FeBDC MOF to Fe_3_O_4_ increases the oxidation current up to 5 times to 14.4 μA as well as reduction current. Also, the oxidation potential of Fe_3_O_4_ is lower than FeBDC; this indicates that Fe_3_O_4_ has better electron transfer compare to FeBDC. To deeply investigate the electrochemical characteristic of Fe_3_O_4_/GCE, we carried out CV measurements at various scan rates in the range of 10–100 mV.s^−1^ with a fixed glucose concentration value of 0.5 mM dissolved in PBS solution of 0.1 mM pH 7.4 with a working voltage range of 0.0–1.0 V.

The possible electrochemical reactions involved in glucose oxidation through the Fe(III)/Fe(II) centers of Fe_3_O_4_ are given below [25,48]:

2Fe(III) + glucose → 2Fe(II) + gluconolactone + H_2_O (1)

gluconolactone + H_2_O → 2H^+^ + gluconate (2)

2Fe(II) → 2Fe(III) + 2e^−^ (3)

The excellent response with the Fe_3_O_4_-modified GCE in the presence of glucose can be attributed to the excellent electrocatalytic nature of Fe_3_O_4_, which mediates the heterogeneous chemical oxidation or reduction of the glucose, while the converted iron oxides can be continuously and simultaneously recovered by electrochemical oxidation or reduction [48]. This back and forth oxidation and reduction of Fe(II)/Fe(III) in Fe_3_O_4_ and glucose are what causesthe CV curve to show the presence of oxidation and reduction currents.

**Figure 7:**
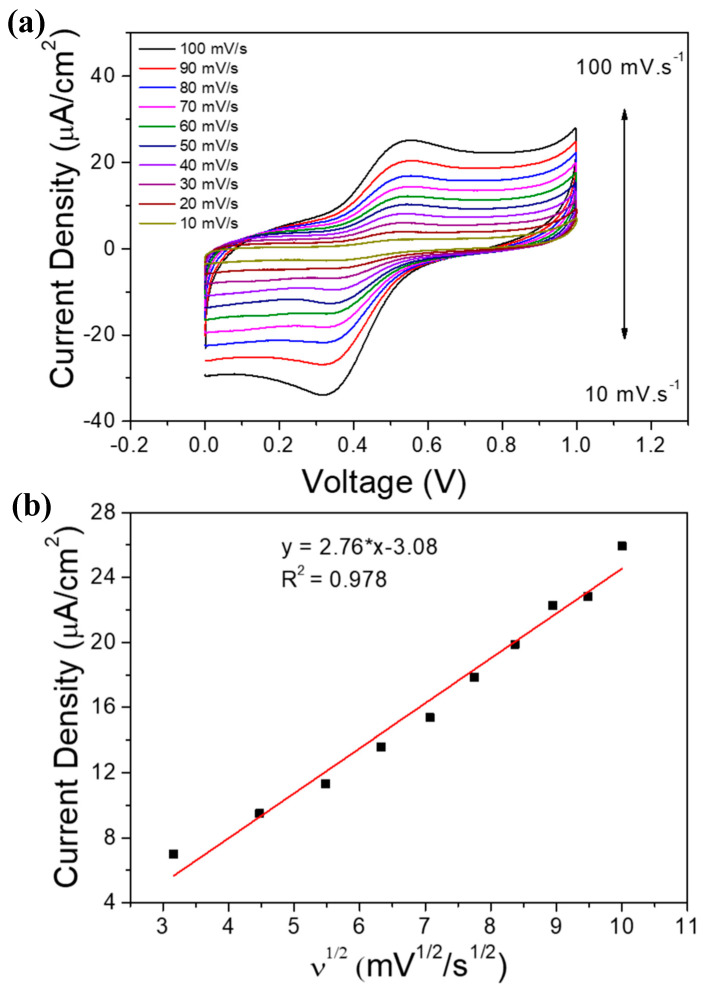
(**a**) CV curves for Fe_3_O_4_/GCE in different scan rate (10, 20, 30, 40, 50, 60, 70, 80, 90, and 100 mV.s^−1^) in 0.5 mM of glucose in 0.1 M of PBS solution pH 7.4; (**b**) linear regression plot for the increasing scan rate.

The relationship between the current density value at the oxidation peak and the square root value of the scan rate was investigated, and the result is shown in Figure 7b. We can see that a higher value of redox peaks current would indicate an increase in scan rate, while the potential for anodic peaks (*E_pa_*) and cathodic peaks (*E_pc_*) experienced positive and negative shifts, respectively. These phenomena are most likely attributable to the internal resistance of the Fe_3_O_4_/GCE. In Figure 7b, the anode peak currents (*I_pa_*) of the Fe_3_O_4_/GCE in 0.1 M of PBS pH 7.4 solution are plotted as a function of the square root of the scan rate (*ν^1/2^*) w. The fitting result reveals a linear trend for *I_pa_* as a function of *ν^1/2^* with a correlation coefficient of 0.978. This result suggests that the electrochemical kinetic mechanism for Fe_3_O_4_/GCE is a diffusion-controlled process. The fact the electrochemical kinetic mechanism for Fe_3_O_4_/GCE is a diffusion-controlled process indicates that the resulting current is a function of analyte’s concentration.

**Figure 8:**
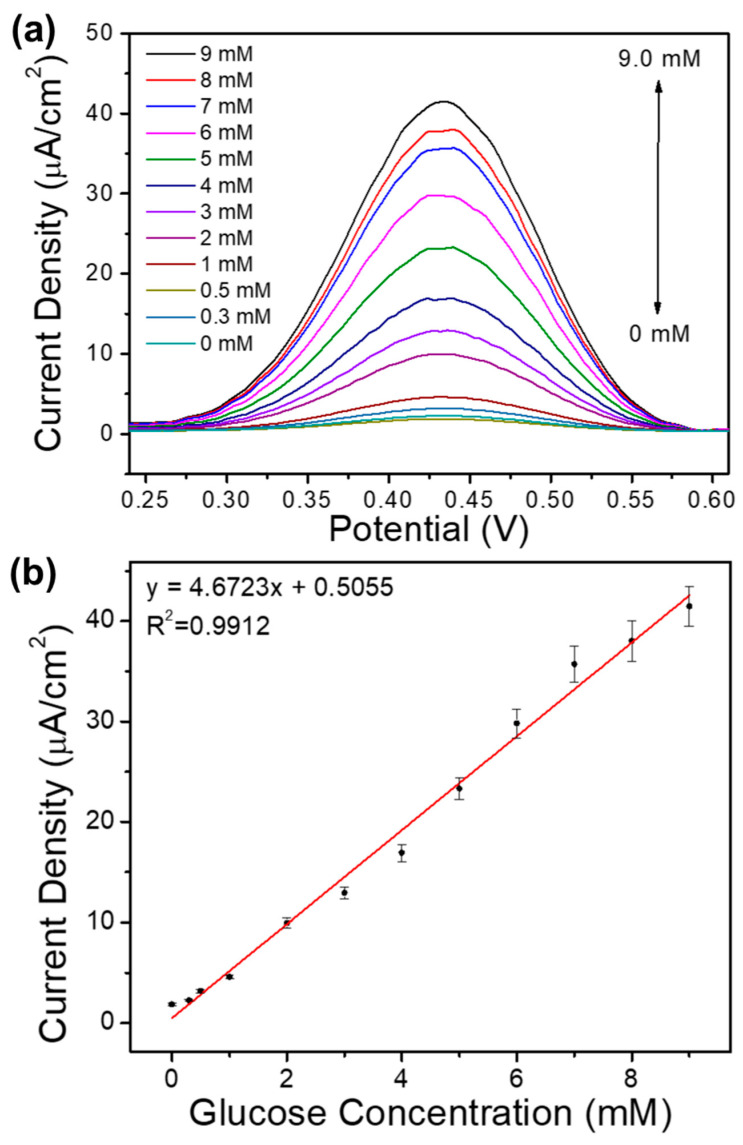
(**a**) DPV measurement of glucose in 0.1 M of PBS pH 7.4 with the variation concentration of (0.0–9.0 mM); (**b**) Linear regression plots between current density and variation in glucose concentration.

As mentioned earlier, the interaction between active surface and glucose in the medium of PBS generated current at a specific voltage. To determine the effect of glucose concentration on the resulting currents, we carried out DPV measurements. DPV measurement is considered as a suitable technique due to it can minimize the effects of capacitive currents that occur in glucose oxidation so that the measured current is pure faradaic current arising from the influence of glucose concentration. The measurement was done by varying the value of glucose concentration in the range 0.0–9.0 mM in PBS solution 0.1 M pH 7.4, and the results are shown in Figure 8a. In contrast, the linear regression plot between values of the resulting current density at a particular concentration to the value of the concentration of glucose contained in the test solution is shown in Figure 8b. The selection of glucose concentration values is based on the category of glucose levels in human blood. The American Diabetes Association defines prediabetes as a condition with a fasting glucose level of 100 to 126 mg/dL or 5.6–6.9 mM [49]. Therefore, variations in concentrations that have a value below the range, within that range, and above that range are chosen to see whether the sample material is potential to be used as glucose-sensing material in the 5.6–6.9 mM of glucose concentration range (prediabetic conditions). The results of DPV measurements show that all the DPV curves in Figure 8a exhibit broad anodic peaks.

Sensitivity is a statistical measure of sensor performance. The sensitivity is then defined as the ratio between the output signal and the measured property [50]. The sensitivity value can be seen from the slope of the concentration to the current regression curve. The linear regression equation of glucose peak is J_peak_ (µA/cm^2^) = 4.67C (mM) + 0.506, where J_peak_ and C represent the current density and concentration of glucose, respectively. From the slope of the function, we obtained a sensitivity value of 4.67 µA mM^−1^.cm^−2^. The performance of the electrochemical sensor (in this case, the resulting current) has a linear relationship with the concentration of glucose with a correlation coefficient of 0.9912. It can be concluded that the linear range of the electrochemical sensor is 0.0–9.0 mM, with the limit of detection (LOD) is as low as 15.70 μM. In this case LOD is defined as a three times of standard deviation (σ) of the blank, 0 mM, divided by slope of the regression line. When compared with the results of the static characteristics of FeBDC, FeBDC pyrolysis increases its performance as a sensor. This increase can be seen from the increased sensitivity, widening of the linearity range, and the decrease in the detection limit. This increase in performance is mainly attributed to the loss of organic ligands which inhibit electron conductivity.

### 3.3. Selectivity

**Figure 9:**
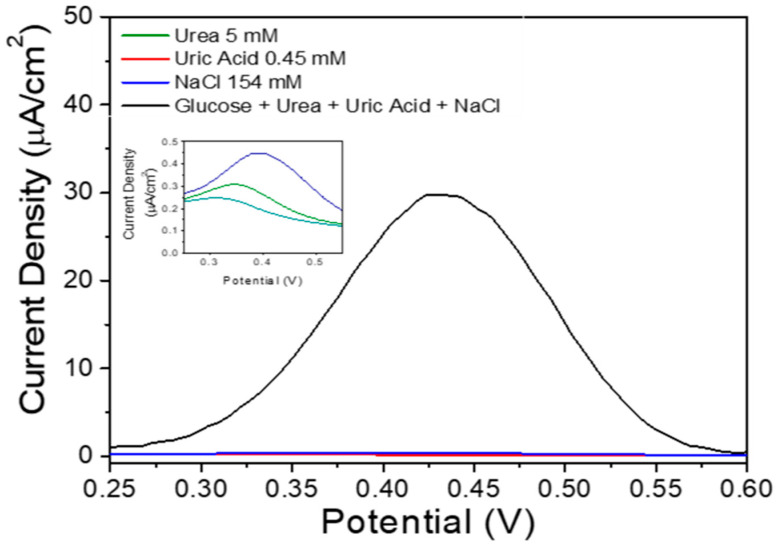
DPV Fe_3_O_4_ voltammogram on 154 mM NaCl, 0.45 mM uric acid, 4.50 mM glucose and 5.00 mM urea in 0.1 M of PBS pH 7.4.

Selectivity testing in this study aims to determine whether the sensor material can detect glucose without being affected by competing species commonly found in the blood. The test was performed using the DPV measurement. The testing process was carried out by giving NaCl, Uric Acid, and urea concentrations according to their respective average concentrations in blood (154 mM; 0.45 mM and 5 mM respectively) and glucose concentrations in the middle of the 3–9 mM detection range, i.e., 6 mM in the 0.1 M ofPBS solution pH 7.4. Figure 9 shows the results of selectivity testing. It appears that the measured current is almost unchanged (29.8 μA to 29.9 μA). This small change in current indicates that the sample material is far more sensitive to glucose than other analytes (Urea, Uric Acid, and NaCl) in the selected potential window and also suggests that the measurement of glucose by FeMOF-derived Fe_3_O_4_ will not be disturbed by other analytes. This result shows that Fe_3_O_4_ is selective against glucose when compared to NaCl, Uric Acid, and Urea.

### 3.4. Stability and Reproducibility

**Figure 10:**
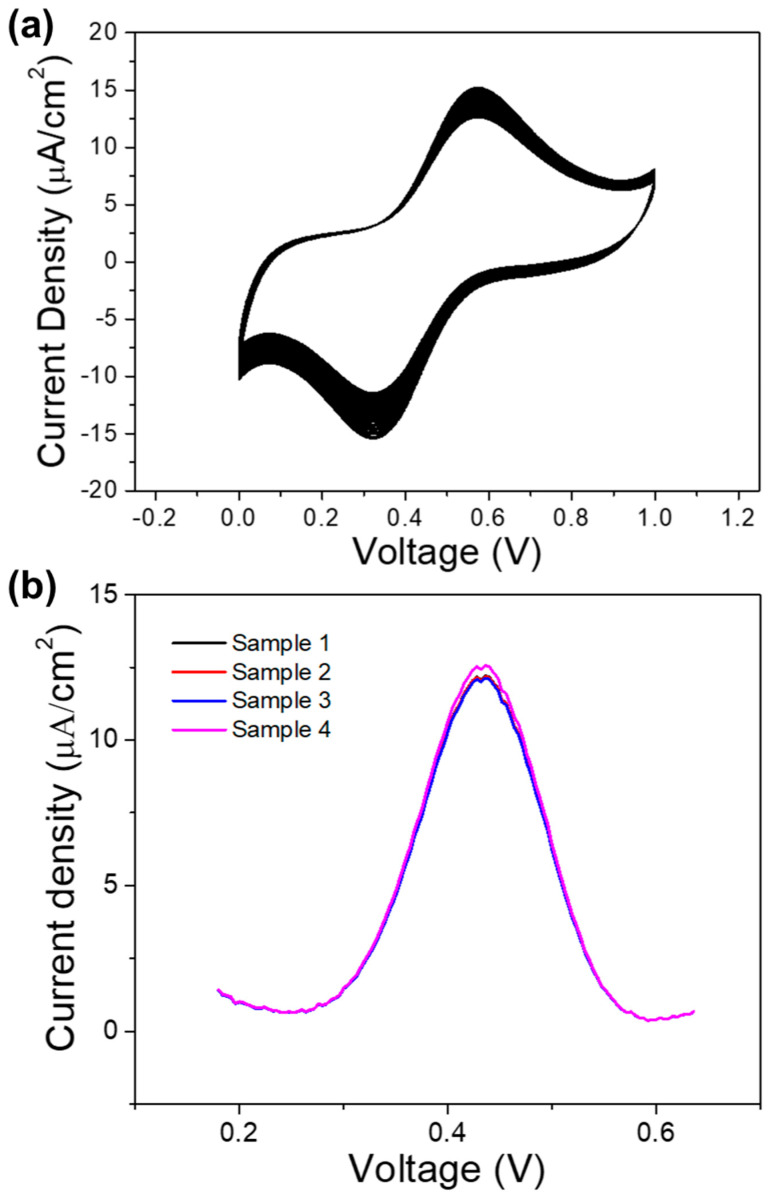
(**a**) CV curves of Fe_3_O_4_-modified GCE in 3 mM glucose at PBS pH 7.4 for 25 cycles (**b**) DPV curves of four different Fe_3_O_4_-modified GCE in 3 mM glucose in 0.1 M of PBS pH 7.4.

The CVs for Fe_3_O_4_ modified GCE in 0.1 M of PBS pH 7.4 containing 3 mM of glucose, were recorded for 25 cycles to find stability. The result is shown in Figure 10a. The current peak in the 25^th^ cycle was found to be 80.25% of the current in the first cycle, indicating that the modified electrode has relatively good stability. Four different GCE were modified with the Fe_3_O_4_, and their response towards the oxidation of 3 mM of glucose was tested to find the reproducibility of the modified electrode, and the result is shown in Figure 10b. The current peaks obtained in the five repeated measurements of four independent electrodes showed relative standard deviance of 1.60%, confirming that the results are reproducible.

### 3.5. Comparison of Sensor Performances

For further understanding the performance of the synthesized Fe_3_O_4_, we compared it with other glucose non-enzymatic sensing material that incorporating Fe_3_O_4_. Different kinds of iron oxide-based non-enzymatic glucose biosensors, reported in the literature, have been listed in Table 2. It can be seen that our work is comparable to other non-enzymatic glucose sensors and offers prominent advantages such as a wide linear range, reasonable sensitivity, and a acceptable detection limit.

## 4. Conclusions

In this work, In this research, we succeeded in synthesizing FeBDC by solvothermal method. The FeBDC was then pyrolyzed to obtain Fe_3_O_4,_ which was used as an active material for non-enzymatic glucose sensors. As synthesis FeBDC has a bulky triangular rodlike structure as a result of coordination between Fe^2+^, DMF, and BDC bridging ligand. However, the transformation from bulky rodlike to the sphere-like Fe_3_O_4_ was observed after pyrolysis was carried out. The performance of non-enzymatic glucose sensor from FeBDC-derived Fe_3_O_4_ material by electrochemical method has sensitivity characteristics of 4.67 µA mM^−1^.cm^−2^, a linear detection range of 0 to 9 mM, a glucose detection limit of 15.70 µM, and good stability and reproducibility. This material is also selective against the interference of urea, NaCl, and uric acid. Therefore, we believe that FeBDC-derived Fe_3_O_4_ has the potential for non-enzymatic electrochemical sensor material to detect glucose levels in the human blood serum analytes.

## Figures and Tables

**Table 1:** Oxidation and Reduction Peak Current and Potential of bare GCE, FeBDC/GCE. Fe_3_O_4_/GCE in 3 mM of glucose and PBS of 0.1 M pH 7.4.

Sample	Oxidation		Reduction	
	Current (µA)	Potential (V)	Current (µA)	Potential (V)
Bare GCE	0.39	0.625	0.28	0.304
GCE + FeBDC	2.98	0.625	2.77	0.304
GCE + Fe_3_O_4_	14.4	0.522	11.99	0.417

**Table 2:** Various non-enzymatic glucose biosensors based on iron oxide.

Material	Sensitivity (μA mM^−1^cm^−2^)	Linear Range	LOD (μM)	Ref
Ni-NPs/rGO	2.5 μA mM^−1^	0.00025–1.2 mM	0.01	[51]
Co_3_O_4_	36.25	0–2.04	0.97	[52]
Co_3_O_4_ nanocrystals	270.9	1.0–7.0	50	[53]
CuO	207.3	0.001 to 6 mM	0.50	[54]
CuO nanowires/PET	-	0–12.0 mM	50	[55]
CuO nano fibers	183.3	0.06–3.0 mM	8	[56]
Octahedral Cu_2_O	241	0.3–4.1mM	128	[57]
ZnO nanorods	2.97	0.1–13.8mM	1000	[58]
Mn_3_O_4_NP/N-rGO	26 μA mM^−1^	1.0–329.5 μM	0.50	[59]
Fe_3_O_4_ nanotube array	9.58	1–5 mM	0.10	[22]
Fe_2_O_3_-P4VP-*co*-PAN	1382.8	2.5 μM–0.58 mM	0.58	[25]
FeOOH nanowires	12.13	0.015–3.0mM	7.8	[60]
FeBDC-derived Fe_3_O_4_	4.67	Up to 9.0 mM	15.70	This work
